# Intranasal oxytocin administration impacts the acquisition and consolidation of trauma-associated memories: a double-blind randomized placebo-controlled experimental study in healthy women

**DOI:** 10.1038/s41386-021-01247-4

**Published:** 2021-12-09

**Authors:** Katharina Schultebraucks, Tolou Maslahati, Katja Wingenfeld, Julian Hellmann-Regen, Julia Kraft, Maureen Kownatzki, Behnoush Behnia, Stephan Ripke, Christian Otte, Stefan Roepke

**Affiliations:** 1 Department of Psychiatry and Psychotherapy, CBF, Charité – Universitätsmedizin Berlin, Corporate Member of Freie Universität Berlin, Humboldt-Universität zu Berlin, and Berlin Institute of Health, Berlin, Germany; 2 Department of Emergency Medicine, Columbia University Irving Medical Center, New York, NY USA; 3 Data Science Institute, Columbia University, New York, NY USA; 4 Department of Psychiatry, NYU Grossman School of Medicine, New York, NY USA; 5 Department of Psychiatry and Psychotherapy, CCM, Charité – Universitätsmedizin Berlin, Corporate Member of Freie Universität Berlin, Humboldt-Universität zu Berlin, and Berlin Institute of Health, Berlin, Germany; 6 Stanley Center for Psychiatric Research, Broad Institute of MIT and Harvard, Cambridge, MA USA; 7 Analytic and Translational Genetics Unit, Massachusetts General Hospital, Boston, MA USA

**Keywords:** Physiology, Biomarkers, Genetics

## Abstract

Intrusive memories are a hallmark symptom of post-traumatic stress disorder (PTSD) and oxytocin has been implicated in the formation of intrusive memories. This study investigates how oxytocin influences the acquisition and consolidation of trauma-associated memories and whether these effects are influenced by individual neurobiological and genetic differences. In this randomized, double-blind, placebo-controlled study, 220 healthy women received either a single dose of intranasal 24IU oxytocin or a placebo before exposure to a trauma film paradigm that solicits intrusive memories. We used a “general random forest” machine learning approach to examine whether differences in the noradrenergic and hypothalamic-pituitary-adrenal axis activity, polygenic risk for psychiatric disorders, and genetic polymorphism of the oxytocin receptor influence the effect of oxytocin on the acquisition and consolidation of intrusive memories. Oxytocin induced significantly more intrusive memories than placebo did (*t*(188.33) = 2.12, *p* = 0.035, Cohen’s *d* = 0.30, 95% CI 0.16–0.44). As hypothesized, we found that the effect of oxytocin on intrusive memories was influenced by biological covariates, such as salivary cortisol, heart rate variability, and PTSD polygenic risk scores. The five factors that were most relevant to the oxytocin effect on intrusive memories were included in a Poisson regression, which showed that, besides oxytocin administration, higher polygenic loadings for PTSD and major depressive disorder were directly associated with a higher number of reported intrusions after exposure to the trauma film stressor. These results suggest that intranasal oxytocin amplifies the acquisition and consolidation of intrusive memories and that this effect is modulated by neurobiological and genetic factors. Trial registration: NCT03031405.

## Introduction

A leading symptom of post-traumatic stress disorder (PTSD) is the intrusive re-experience of a traumatic event [1]. Intrusive memories are defined by recurrent involuntary distressing recollections or nightmares of the experienced trauma [2]. While intrusive memories after traumatic events are part of a normal adaption process [3], their frequency [4, 5], vividness, and perceived distress are predictive of PTSD [6–9].

Biological factors that influence the development of these intrusive memories are not sufficiently understood. Heterogeneous associations have been reported between PTSD and hormonal and neuroendocrine dysregulation, such as in the hypothalamic-pituitary-adrenal (HPA) axis and the noradrenergic system [10, 11]. Biomarkers associated with psychosocial stress, such as cortisol, salivary α-amylase (sAA), and heart rate variability (HRV), have been associated with intrusive memories after trauma [12–15].

The oxytocin system is thought to play a crucial role in the development of PTSD [16, 17]. Oxytocin was initially presumed to have anxiolytic effects [18, 19]. Studies focusing primarily on these anxiolytic effects found that exogenous oxytocin reduces activation of the HPA axis, which is needed to adapt to high stress such as traumatic events [20–23]. Oxytocin dampened cortisol reactivity to a laboratory stress task in patients with PTSD and comorbid alcohol use disorder who have high baseline cortisol levels [24]. Oxytocin has also been found to increase pro-social abilities, such as showing empathy [25], recognizing emotions in facial expressions [26, 27], and remembering social stimuli [28]. In contrast, oxytocin was also found to increase the subjective perception of stress after a social stress test [29], the startle response to threat [30], and distrust [31]. It also triggered aggressive reactions to threads [32, 33] and increased envy and gloating in a competitive task [34]. Recently, the social salience hypothesis of oxytocin has explained these contradictory findings [35]. This theory states that oxytocin increases sensitivity to salient cues in the environment [36, 37]. In this way, oxytocin reduced functional connectivity between the amygdala and pre-frontal cortex when administered before exposure to the stressor and increased flashback intensity after the trauma script was observed [38]. Further, exogenous oxytocin facilitated fear learning [39], increased alertness to threat [29, 40], and enhanced emotional learning [41, 42]. In agreement with these observations, other studies provide preliminary evidence that repetitive administration of exogenous oxytocin after exposure to a trauma stressor reduced intrusions and PTSD symptoms [43, 44].

Genetic factors also influence the susceptibility to PTSD [45, 46]. The single nucleotide polymorphism (SNP) rs53576 in the oxytocin receptor gene was found to be associated with PTSD symptoms together with attachment style [47] or a negative social environment [48]. The rs53576 SNP has also been found to modulate intranasal oxytocin effects [49, 50], while another polymorphism of the oxytocin receptor gene (rs2254289) has been associated with general psychopathology [51].

Individual SNPs cannot fully explain variations in disease risk [52–54]. Genome-wide approaches including polygenic risk scores (PRS) are needed to investigate the cumulative impact of genetic risk variants [46]. PTSD onset and severity have been associated with a higher polygenic burden for PTSD, and these effects vary by sex [45, 46]. Moreover, psychiatric disorders are highly polygenic and have a shared molecular architecture [55, 56]. The latest genome-wide association study (GWAS) of PTSD revealed significant genetic correlations with general psychopathology, cross-disorder, and other psychiatric disorders [46]. This is not an unexpected finding given that mood disorders and other mental disorders are frequent comorbidities in PTSD and that several PTSD symptoms overlap with symptoms of other psychiatric disorders such as major depressive disorder (MDD) [57]. It is worthwhile exploring cross-trait polygenic associations with PTSD symptoms.

This study examined the effect of exogenous oxytocin on the acquisition and consolidation of trauma-related memories, which has, to the best of our knowledge, not been investigated yet. Based on the social salience hypothesis [35], we hypothesized that exogenous stimulation of the oxytocin system with a single intranasal dose of oxytocin at the time of acquisition and consolidation of a stressful event would induce more intrusive memories over the following four days than administration of placebo would. As the etiology of PTSD is complex and heterogenic [58], it is important not to look for solitary predictors, but rather to include a multitude of potential predictors. Machine learning approaches can investigate multiple predictors and their complex interactions [59] and has been used to identify biomarkers of stress pathologies after trauma [60–62] including the combination of multiple PRS [63]. This study used a data-driven approach to investigate the complex relationships between intrusive memories and oxytocin, and to examine genetic variables and neuroendocrine and neurophysiological markers associated with acute psychosocial stress.

## Materials and methods

The randomized, double-blind, placebo-controlled study was conducted at the Department of Psychiatry and Psychotherapy, Campus Benjamin Franklin, Charité — Universitätsmedizin Berlin. The study was approved by the local ethics committee of Charité — Universitätsmedizin Berlin (EA4/144/16). All participants gave written consent after being informed about the study at least 24 h before the study started. The testing started at the same time (2 pm) to account for differences in cortisol levels due to the circadian rhythm [64]. Test conditions have been previously described [15].

### Participants

**Fig. 1:**
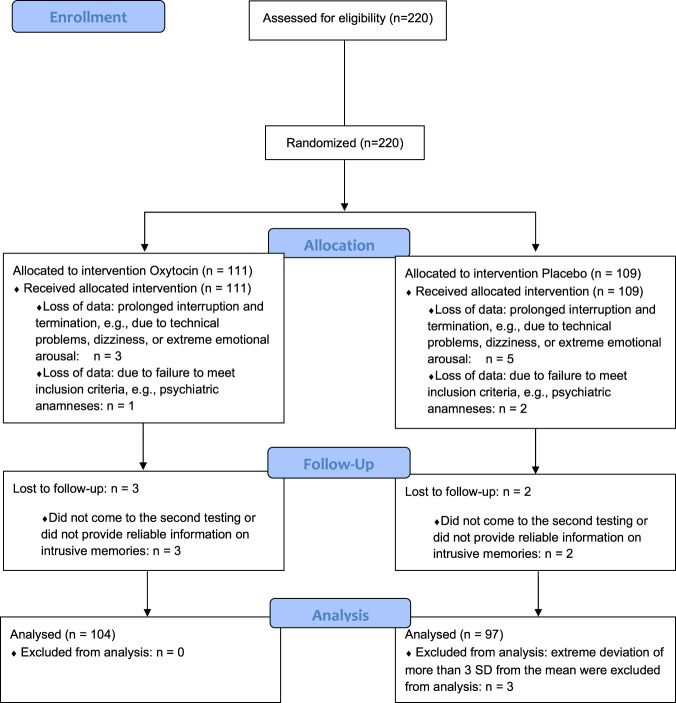
Flow Diagram. Flow chart showing the inclusion and exclusion of participants during the course of the study.

We recruited 220 healthy female university students (Fig. 1) via university email lists or public postings. Because the protagonist (i.e., victim) in the trauma film paradigm is female and because oxytocin has sexually dimorphic effects [65], only female subjects were included. Mental and physical health-related aspects were assessed before participation to verify eligibility criteria as described previously [15] and in the Supplemental Information. Participants not using hormonal contraception were tested during their luteal cycle phase to rule out effects of fluctuating hormones, which has been shown to influence endogenous oxytocin [66] and intrusion formation [67, 68].

A HCG ULTRA pregnancy test was used to make sure participants were not pregnant. All participants were between the age of 18 and 30 years, spoke native-level German, and received a reimbursement of 40€. Four weeks after the experiment, participants were contacted over the telephone to ensure full recovery from the adverse film and were offered psychological care, in case they experienced any ongoing distress after the trauma film paradigm. None of the participants needed psychological care and had to make use of the offer.

A priori power analysis indicated that a sample size of 200 would be needed to detect small to moderate effects (Cohen’s *d* = 0.4, *α* = 0.05, power = 0.8). To factor in possible dropouts, 220 participants were enrolled.

### Experimental phase

The participants were given either 24IU oxytocin (nasal spray, Syntocinon®) or a placebo preparation (sodium chloride nasal spray) once before watching the stress-inducing film clip (analog trauma). Participants were randomly allocated to the groups using Research Randomizer [69]. Both, the participants and the examiner were blinded to treatment allocation and the examiner did not take part in the randomization process. The placebo preparation and the oxytocin nasal spray looked identical to ensure the double-blind design. The primary outcome was measured and the statistical analysis was performed with participants and examiners blinded.

Considering peak levels of oxytocin [70], the nasal spray was given 40 min before the trauma film was shown. Potential effects of oxytocin and the trauma film on salivary cortisol levels, sAA activity, and HRV were assessed seven times during the study: at baseline, directly before the trauma film, and five times every 15 min after the film had ended. At the end of the session, participants were told how to fill out the diary over the next four days.

Descriptions of the analog trauma exposure, the intrusion diary, psychometric and salivary assessment, and measurement of HRV have been published [15] and are presented in the Supplemental Information. Details on genomic data processing and polygenic scoring are presented in the Supplemental Information, Supplemental Figs. 1–3 and Supplemental Table 1.

### Statistical analysis

We used a Chi-square test and Student’s *t*-test or the non-parametric Mann–Whitney *U*-test to compare differences in sample characteristics between both groups. Mixed-design analyses of variance with time as the within-subject factor and treatment (oxytocin vs. placebo) as the between-subject factors were used to analyze changes in salivary cortisol, sAA, and HRV.

#### Mean group differences: average treatment effect (ATE)

To assess the primary outcome of the study, we used the Student’s *t*-test to find differences in the mean number of intrusions per treatment assignment (Cohen’s *d* including 95% CI). We also estimated ATEs based on targeted maximum likelihood learning [71] of the generalized random forests (GRF) approach described below [72].

#### Heterogeneous treatment effects (HTE)

If intervention effects (ATE) are not homogeneous within treatment groups but vary with differences in covariate space, the mean is not the best representation of heterogeneity in treatment effects per group. We examined which covariates explain heterogeneity of the effect of oxytocin on the number of intrusive memories. Multiple biological (i.e., noradrenergic system or HPA axis activity) and genetic characteristics are plausible covariates for moderating the intervention effect per group based on prior literature, but the best functional form (e.g., linear association) to model these potential effects is unknown. The new GRF statistical modeling approach [72] is a principled statistical method for systematically estimating heterogeneity in treatment effects in experimental studies in a data-driven way [73]. We used GRF to run a non-parametric omnibus hypothesis test about the magnitude of differences in treatment effects across subsets of the population [72] using recursive partitioning of the data extending the classical random forest algorithm [74, 75]. We tested for heterogeneity using qualitative descriptions of “responders” vs. “non-responders” by splitting the sample on the median conditional average treatment effect (CATE). We used the heteroskedasticity-consistent test of calibration to detect heterogeneity [72, 76]. Details on data preprocessing and hyperparameter tuning are presented in the Supplemental Information.

#### Variable importance

We examined which variables best predict variance in the intervention effects by ranking the covariates in order of importance. The ranking is calculated as the sum of how often a given covariate is split at each depth of the forest. The sum is weighted so that early splits (low forest depth) are more important than late splits. Variables are considered “more important” if the variable is more frequently used for the first splits across all decision trees that are grown in the random forest [72, 77]. We estimated the best linear projection by fitting the CATE as a linear function of the “out-of-bag” random forest estimates [76, 78] to understand whether and how the intervention effect of oxytocin on the number of intrusions depends on differences in covariates. The “best” linear projection is the one covariate (or a set of covariates) that significantly predicts the group effects as a linear relationship (standard errors [SE] and 95% CI).

## Results

Of the *n* = 220 enrolled participants randomized either to oxytocin or placebo, we included *n* = 201 participants in the final analysis (Fig. 1). The 201 analyzed participants did not significantly differ from the 19 excluded participants regarding the characteristics presented in Table 1, apart from the BDI-II score (Supplemental Table 2). As shown in Table 1, there were no significant differences in characteristics between the oxytocin (*n* = 104) and placebo group (*n* = 97). Supplementary Fig. 5 illustrates the longitudinal development of salivary cortisol, sAA activity, and HRV before and after the administration of oxytocin vs. placebo and before and after the trauma film was shown. There was a significant change in cortisol levels (*F*(2.06, 410.16) = 9.04, *p* ≤ 0.001), sAA activity (*F*(4.423, 880.19) = 13.59, *p* ≤ 0.001), and HRV (*F*(4.63, 795.95) = 14.11, *p* ≤ 0.001) as the study progressed. There was no significant difference in cortisol levels (*F*(1,199) = 0.67, *p* = 0.41), sAA activity (*F*(1,199) = 0.14, *p* = 0.71), or HRV (*F*(1,172) = 0.02, *p* = 0.88) between the oxytocin and placebo groups.

**Table 1:** Sample characteristics.

Characteristics	Oxytocin (*n* = 104) *M* (SD) or *n*	Placebo (*n* = 97) *M* (SD) or *n*	Statistics
Age	23.22 (3.39)	22.70 (3.14)	*U* = 4623.50, *Z* = −1.03, *p* = 0.31
Intake of oral contraceptives	37	41	$\chi$^2^(1) = .95, *p* = 0.33
Current smoker	27	34	$\chi$^2^(1) = 2.13, *p* = 0.15
BMI	21.88 (2.33)	21.73 (2.7)	*U* = 4751.50, *Z* = −0.71 *p* = 0.48
CTQ	30.72 (6.34)	31.24 (7.39)	*U* = 4987.00, *Z* = −0.14, *p* = 0.89
STAI-T	32.27 (5.97)	33.63 (6.7)	*U* = 4415.50, *Z* = −1.53, *p* = 0.13
BDI-II	3.60 (4.07)	4.18 (3.95)	*U* = 4319.50, *Z* = −1.77 *p* = 0.08
ERQ reappraisal	29.41 (5.11)	29.04 (5.07)	*U* = 4911.00, *Z* = −0.32, *p* = 0.75
ERQ suppression	11.32 (4.13)	10.89 (3.89)	*U* = 4723.00, *Z* = −0.78, *p* = 0.43
Participants who had seen the film before	7	9	$\chi$^2^(1) = 0.45, *p* = 0.51

*M* mean, *SD* standard deviation, *BMI* body mass index, *CTQ* Childhood Trauma Questionnaire, *STAI-T* state-trait anxiety inventory-trait subscale, *BDI-II* Beck depression inventory-revised, *ERQ* emotion regulation questionnaire (subscales reappraisal and suppression).

### Mean group differences

**Fig. 2:**
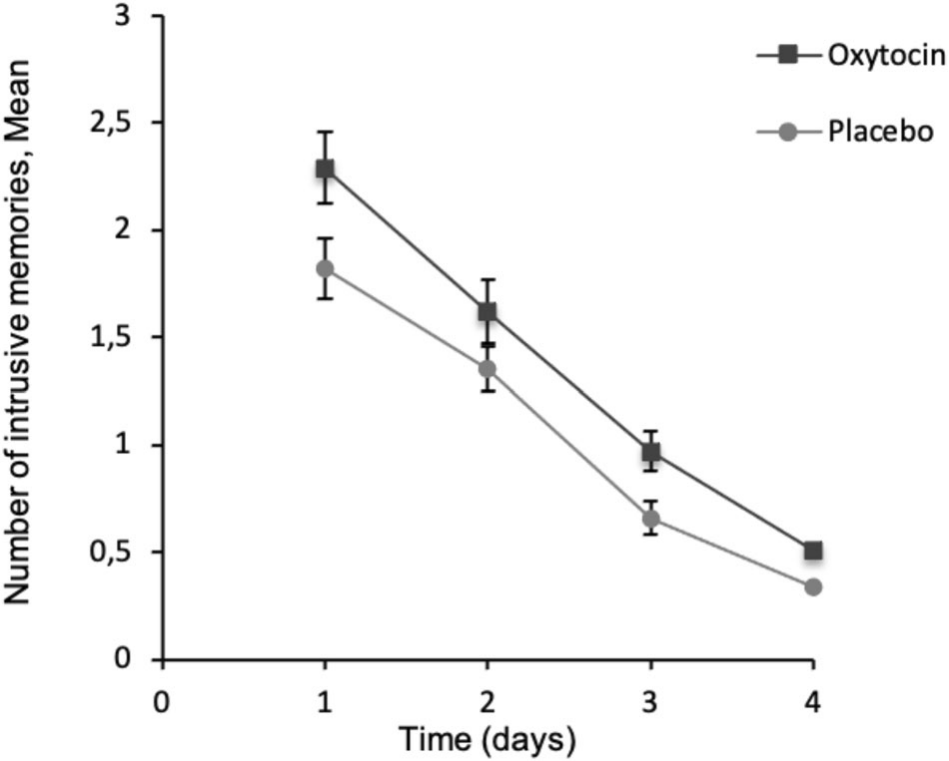
Number of intrusive memories in the oxytocin and placebo group over 4 days. Points are means, with standard errors represented by vertical bars.

A two-sided Welch’s *t*-test (*t*(188.33) = 2.12, *p* = 0.035, Cohen’s *d* = 0.30, 95% CI 0.16–0.44) showed that the group receiving oxytocin reported significantly more intrusive memories (mean = 5.39 ± 4.96 SD) than the placebo group did (mean = 4.09 ± 3.62 SD). Figure 2 depicts the number of intrusive memories over 4 days.

### Average treatment effect

The ATE was 1.25 (SE = 0.61) based on the targeted maximum likelihood learning using the GRF. Following the approach suggested by Athey and Wager [79], we further tested for heterogeneity in subgroups depending on the covariates using a median-split into “high” vs. “low” out-of-bag CATE estimates. Within the oxytocin group, participants with an estimated “high” oxytocin effect (i.e., above the median) showed 2.52 (SE = 0.82) more intrusions than the “low” group did (0.01; SE = 0.90). There was a significant effect of oxytocin vs. placebo in this “high effect” subgroup (t(88.17) = 3.16, *p* = 0.002, Cohen’s *d* = 0.60, 95% CI 0.45–0.75), which was more pronounced than the overall effect, indicating that oxytocin did not have a homogeneous effect in our sample. The heterogeneity of the treatment effect was underscored by the significant omnibus test for the differential forest prediction (estimate = 0.58, SE = 0.32, *t* = 1.80, *p* = 0.037) indicating that the random forest captures heterogeneity in the intervention effect [76]. The overlap assumption was fulfilled, i.e., the estimated propensity scores were not close to one or zero (Supplemental Fig. 4).

### Variable importance

**Fig. 3:**
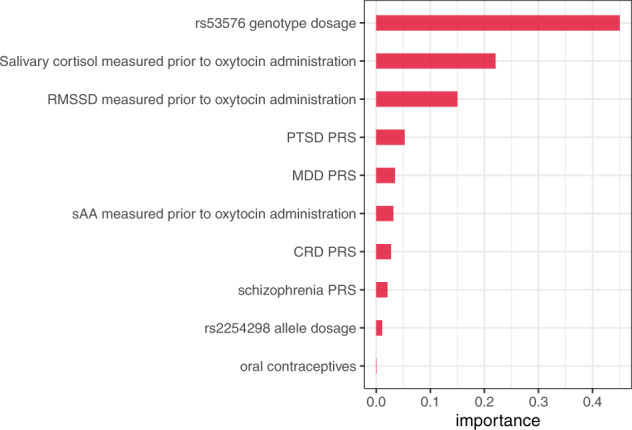
Variable importance ranking of the covariates that are most informative in the random forest model at predicting differences in the treatment effect, i.e., to determine factors associated with differences in how the administration of oxytocin affects self-reported intrusive memories after exposure to a trauma film. *RMSSD* root mean square of successive differences, *PTSD PRS* post-traumatic stress disorder polygenic risk score, *MDD PRS* major depressive disorder polygenic risk score, *sAA* salivary α-amylase activity, *CRD PRS* cross-disorder polygenic risk score.

The variable importance ranking for predicting the CATE of oxytocin administration on the number of intrusive memories is shown in Fig. 3. The highest rank variables were rs53576 allele dosage, salivary cortisol, root mean square of successive differences (RMSSD) prior to oxytocin administration, and PTSD PRS. The moderating effect of rs53576 is further discussed in the Extended Results in the Supplemental Material and in Supplementary Figs. 6 and 7.

### Poisson regression

We fitted a Poisson regression using the five most important features included in the CATE analysis describing the heterogeneity of the oxytocin effect (Fig. 3). We used a stepwise approach to examine how those features predict the number of intrusive memories. Treatment, PTSD PRS and MDD PRS remained significant predictors of the number of intrusive memories (Table 2 and Supplemental Fig. 8). For both treatment conditions (oxytocin vs. placebo), there was a positive association between the number of intrusions and the PTSD PRS (estimate = 2.21, SE = 1.08, *z*-value = 2.05, *p* = 0.04) and MDD PRS (estimate = 2.30, SE = 1.09, *z*-value = 2.12, *p* = 0.03) (Supplemental Fig. 9, panel a and b).

**Table 2:** Stepwise Poisson regression.

	Estimate	Std. error	*z*-value	*p*-value
(Intercept)	−0.82	1.08	−0.76	0.45
Treatment (oxytocin vs. placebo)	0.27	0.07	4.07	4.73e−05***
RMSSD prior to oxytocin administration	−0.68	0.39	−1.74	0.08
PTSD PRS	2.21	1.08	2.05	0.04*
MDD PRS	2.30	1.09	2.12	0.03*

*RMSSD* root mean square of successive differences, *PTSD PRS* post-traumatic stress disorder polygenic risk score, *MDD PRS* major depressive disorder polygenic risk score. Significance thresholds: 0.001 = ***, 0.01 = **, 0.05 = *. Deviance residuals: Min. −3.56, 1Q = −1.75, Median = −0.53, 3Q = 0.91, Max. = 5.91, AIC: 1331.3.

## Discussion

In this study, we investigated the impact of exogenous oxytocin administration on the acquisition and consolidation of intrusive memories after watching a trauma film. Healthy young females reported more intrusive memories after receiving 24 IU intranasal oxytocin than after receiving placebo before watching the film. The effect size in this highly controlled setting and homogeneous, healthy sample was comparable to those in previous behavioral studies using a single dose of oxytocin (reported mean effect size for healthy subjects of Cohen’s *d* = 0.28 [80]).

As hypothesized, we observed a higher number of intrusions after exogenous stimulation of the oxytocin system compared to placebo. Consistent with the social salience hypothesis [35], intranasal oxytocin administration facilitates the encoding and consolidation of salient trauma-related memories, thereby increasing the number of self-reported intrusions after the trauma film.

Our results match previous studies that reported increased subjective perception of stress [29], startle response to threat [30], and distrust [31] after oxytocin administration. In prior studies, exogenous oxytocin administration increased the intensity of intrusive memories after a trauma script [38], facilitated fear-learning [39], enhanced alertness to threat [29, 40], and increased emotional learning [41, 42]. In contrast to our findings, a recent study with a smaller sample size found no effect of oxytocin on the number of subsequent intrusive memories; however, oxytocin was associated with higher intrusion-related distress mediated by peri-traumatic stress during trauma recording [81]. Furthermore, previous research has shown that the basal endogenous oxytocin levels measured in serum prior to deployment do not seem to predict combat-related PTSD after deployment [82]. The effect of oxytocin on subsequent PTSD symptoms seems to depend on the timing and frequency of oxytocin administration. Administration of oxytocin directly after exposure to stress induced a short-term increase in traumatic memories while chronic administration of oxytocin after exposure to stress had anxiolytic effects [83]. In line with these studies are preliminary findings that repeated administration of oxytocin can reduce PTSD symptoms in patients with acute distress after trauma [43], and in patients with PTSD [44]. Another study differentiated the effect of repeated post-trauma oxytocin administration and further supports the notion that oxytocin enhances the salience of the social signal [84]. Oxytocin administration led to decreased intrusions in participants with strong trauma disclosure, an effect that was not found in participants with weak trauma disclosure [84].

We wanted to investigate the heterogeneity among the noradrenergic system, HPA activity, polygenic risk, and genetic polymorphisms of the oxytocin receptor; to this end, we performed an exploratory analysis using machine learning (i.e., general random forests). We found that the effect of the intervention (i.e., intranasal oxytocin) on the outcome (i.e., self-reported number of intrusions) differed significantly between participants with different covariate profiles (e.g., differences in SNP rs53576, especially with GG polymorphism, cortisol, HRV, and PTSD PRS; Fig. 3). The algorithmic complexity of the general random forest allows for non-linear and higher-order interactions in the covariate space, so the significant dependence of the intervention effect on the covariate space that we observed is not easy to interpret directly. To complement the CATE findings, we also examined the direct effect of the covariates on the number of intrusive memories using a stepwise Poisson regression. This analysis indicated that, in addition to oxytocin administration, PTSD PRS and MDD PRS are significant predictors of the number of intrusive memories. These results provide evidence that the self-reported number of intrusions after a trauma film in young healthy females is influenced by oxytocin, PTSD PRS, and MDD PRS; therefore, these variables should be investigated further in future research.

### Neurobiological and genetic factors influencing the oxytocin effect

We found preliminary evidence that the oxytocin receptor SNP rs53576 is associated with the effect of oxytocin on intrusive memories, in agreement with previous findings that rs53576 influences the effect of oxytocin [49, 50]. However, there are no meta-analytic data to confirm this effect, due to a lack of comparable findings [66]. The current results should be replicated in future studies to corroborate or discount these exploratory findings.

We also found that PTSD PRS impacted the effect of oxytocin on the development of intrusive memories. Previous studies have shown that the PTSD PRS affects the development, onset, and severity of PTSD [45, 46]. Here, we extend these findings by showing that the PTSD PRS can also predict the number of intrusive memories in healthy young women and that the PTSD PRS also influences the effect of oxytocin.

We observed that HRV and cortisol levels before oxytocin administration influence the effect of oxytocin on the number of intrusive memories. In accordance with these results, previous studies have shown that lower baseline HRV predicts more intrusive memories [14, 15]. While exogenous cortisol administration did not affect intrusions in one previous study [12], endogenously increased cortisol levels did predict intrusive memories [15]. Regarding the association of oxytocin and cortisol, previous research has suggested that oxytocin dampens the reactivity of the HPA axis in secure environments [21], but increases the startle response to threat [30]. Furthermore, earlier studies found a positive association between cortisol and oxytocin levels, which may reflect cortisol-induced oxytocin release [85, 86]. The current study is the first to examine how baseline cortisol levels and baseline HRV affect how oxytocin administration influences intrusive memories. This builds on previous findings that baseline HRV and endogenous cortisol levels affect intrusive memories [15, 87], and that oxytocin levels are associated with altered cortisol levels [85, 86].

### Neurobiological and genetic factors influencing the number of intrusive memories

We showed that the number of intrusive memories is influenced by the PTSD PRS and the MDD PRS. A higher PTSD PRS and MDD PRS was associated with more intrusions, independent of whether the participant received oxytocin or placebo. The association we observed between PTSD PRS and the number of intrusions was expected as intrusive memories are a hallmark symptom of PTSD and a higher PTSD PRS increases the likelihood of developing PTSD [45, 46]. Similarly, the association between MDD PRS and intrusive memories may be explained by similarities between depression and anxiety disorders. On the one hand, the positive association between the MDD PRS and intrusive memories may reflect biological pleiotropy, where one risk variant affects two phenotypes [88]. On the other hand, the association may reflect a polygenic overlap between PTSD and MDD, as reported previously [89].

On a trend level, lower baseline HRV was associated with more intrusions. This adds to previous findings that low baseline HRV predicts the development of intrusions after trauma [14, 15] and PTSD [90, 91].

#### Strength and limitations

A strength of this double-blinded randomized placebo-controlled study is the internal validity due to carefully defined inclusion criteria, high experimental control, pre-registration with pre-defined rules of conduct during the measurements, and the stringent operationalization of intrusive memories. Measuring HRV with a heartbeat monitor is a reliable and cost-effective and time-effective alternative to measuring HRV with an electrocardiogram [92]. Endogenous oxytocin concentrations are influenced by the menstrual cycle [93] and hormonal contraceptive intake [94], so we controlled for the use of hormonal contraceptives and tested participants during their luteal phase.

Some limitations of this study should be considered. Intrusions were examined following a relatively mild stressor that may differ from real traumatic events that cause PTSD. Therefore, it is not certain if conclusions can be drawn about the formation of intrusions in patients with PTSD based on the current data. Moreover, the assessment of intrusions by self-report measures poses the risk of response bias [95]. Nevertheless, trauma film paradigms are valid instruments for investigating pre- and peri-traumatic PTSD factors [96]. Certain features of initial intrusive memories have been shown to predict a PTSD diagnosis, such as their frequency [97], “here and now” quality, related distress, and “lack of context” [98, 99]. Moreover, individuals with PTSD experience intrusions more vividly [100], so we defined intrusions by frequency, modality (image, thought, or both), content, liveliness, and degree of stress (0 = “not at all” to 5 = “very strong”) according to Holmes and Bourne [101]. Another promising method to investigate factors influencing the development of PTSD is the virtual reality paradigm [102–104]. The effect of oxytocin on intrusive memories that we report here could be further investigated using a virtual reality paradigm.

Due to the sexually dimorphic effects of oxytocin [65, 66], only female participants were included to increase internal validity. Further, the trauma film was tailored to female participants [105]. Therefore the current results are restricted to women and may not be transferred to men. Sexually dimorphic effects of oxytocin may be due to sex-specific differences in oxytocin plasma concentrations [106] and due to differences in oxytocin expression in different brain regions between men and women [107]. Because of strict exclusion criteria, the sample consisted of a homogeneous group of highly educated healthy women only. Even though female sex and younger age are risk factors for PTSD [1], these findings need to be extended to a more heterogeneous sample, as they may not be generalizable to more vulnerable populations, such as individuals with previous traumatic experiences. A further limitation of the study is that we did not conduct a treatment check for oxytocin. Finally, the time of administration and the effective period of oxytocin do not discriminate between the effects of oxytocin during encoding or consolidation of the trauma film on the formation of intrusive memories.

## Conclusions

We found that increased exogenous oxytocin stimulation during trauma is associated with an increased number of intrusions in young healthy females, consistent with the social salience hypothesis. Genetic variation and neurobiological systems modulate the effect of oxytocin on the development of intrusive memories. We found that oxytocin increased the number of intrusions depending on the rs53576 SNP, salivary cortisol, HRV prior to oxytocin administration, and the PRS for PTSD and MDD. We showed that oxytocin and PTSD and MDD PRS are related to the development of intrusions. In summary, this randomized placebo-controlled trial showed how oxytocin and PTSD vulnerabilities influence the development of subsequent intrusions in healthy young women. Future studies should investigate this oxytocin effect further to better understand and potentially prevent the development of trauma sequelae.

## Funding and disclosure

Katharina Schultebraucks was supported by the German Research Foundation (SCHU 3259/1-1) and the project was funded by a Grant from Stiftung Charité dedicated to Stefan Roepke (BIH_PRO_280). Christian Otte receives honoraria for lectures and/or scientific advice from Ferring, Janssen, Lundbeck, SAGE Therapeutics, Fortbildungskolleg, Limes Klinikgruppe, and Medical Tribune. He also receives research funding from the German Research Foundation (OT 209/7-3; 14-1, EXC 2049), the European Commission (IMI2 859366), the German Federal Ministry of Education and Research (KS2017-067), Berlin Institute of Health (B3010350), and Janssen. Stefan Roepke receives honoraria for lectures and/or scientific advice from Boehringer Ingelheim and Stillachhaus. He also receives research funding from the German Research Foundation, the German Federal Ministry of Education and Research, Innovationsfond, Berlin Institute of Health, and Bionorica SE. All other authors declare no potential conflict of interest.

## Supplementary information

Supplemental Material

## Data Availability

The data is available upon reasonable request but for research purposes only. Please send requests to the principal investigator Stefan Roepke (Department of Psychiatry and Psychotherapy, CBF, Charité – Universitätsmedizin Berlin).
