# Supplementary material for: Intranasal oxytocin administration impacts the acquisition and consolidation of trauma-associated memories: a double-blind randomized placebo-controlled experimental study in healthy women
Source: Neuropsychopharmacology. 2021 Dec 9;47(5):1046–54. doi: 10.1038/s41386-021-01247-4 (PMC8938422; doi:10.1038/s41386-021-01247-4)
Supplement: Supplementary file 1 — Supplemental Material [file 41386_2021_1247_MOESM1_ESM.docx]

**Supplemental Information**

**Extended Methods and Materials**

***Participants***

Exclusion criteria were: (1) any physical illness, (2) any medication intake (except hormonal contraceptives) as these may interfere with oxytocin effects and physiological parameters, (3) a Body Mass Index (BMI) > 30 [1, 2], (4) pregnancy or lactation, (5) excessive sport [3], (6) excessive nicotine consumption [4], (7) blood phobia (because the trauma film contained scenes of violence and bleeding injuries), (8) history of trauma exposure, and (9) any lifetime or current Axis 1 disorder, as verified by the screening questionnaire of the German version of the Structured Clinical Interview for DSM-IV (SCID- I) [5].

**Psychometric assessment**

The Beck Depression Inventory-Revised (BDI-II) [6] was used to capture potential depressive symptoms. A BDI-II score > 13 indicated clinically relevant depressive symptoms [6] and led to exclusion. The Childhood Trauma Questionnaire [7] and the Traumatic Antecedents Questionnaire [8] were used to detect any childhood trauma that may impact the noradrenergic responses to stress [9] and may increase the risk of post-traumatic stress dirsorder (PTSD) after a secondary trauma in adulthood [10]. The Emotion Regulation Questionnaire [11] was used to assess emotion regulation strategies because these can affect the formation of intrusions [12]. The State-Trait Anxiety Inventory [13] was used to determine differences in anxiety because pre-trauma trait anxiety is positively associated with PTSD [14]. These questionnaires were used to detect and exclude psychopathology in participants and to control for possible influences on the formation of intrusive memories.

***Trauma film***

A well-established trauma film paradigm [15-17], which reliably triggers intrusive symptoms [17-19] was presented to the participants in a dark room on a 2x2.5m screen. The sound was played through headphones. The film clip is an excerpt (13:47 minutes) from the commercial film *Irreversible* by Gaspar Noë [20]. The scene depicts a woman being attacked, raped, and physically abused by a stranger in a pedestrian underpass. A female researcher was present during the exposure to the analog trauma, to verify that participants watched the scene without closing their eyes or taking off the headphones.

***Intrusion diary***

Intrusive memories were collected over the following four days after the assessment using a paper-pencil diary, which has also been used in previous studies [18, 19]. A daily text message was sent at 9 p.m. to remind participants to transfer inscriptions from their paper pencil diary to an identical online diary to assure participation and to avoid data loss. Participants were instructed to record any memory of the film as soon as it occurred. According to Holmes, et al. [21], memories were specified by frequency, modality (image, thought, or both), content, liveliness, and degree of stress (0 = “not at all” to 5 = “very strong”). Memories were defined as intrusions if they popped up spontaneously and involved imagery with vividness and distress > 1 [22, 23].

***Salivary assessment***

To measure free cortisol and sAA in saliva, we collected saliva samples in salivettes (blue cap, Sarstedt, Germany), which were immediately stored at -80 °C (-112 °F) until biochemical analysis. Biochemical analyses were performed in the Neurobiology Laboratory of the Department of Psychiatry and Psychotherapy, Charité – Universitätsmedizin Berlin. As previously described in detail [18], salivary cortisol was analyzed using a homogeneous time-resolved fluorescence resonance energy transfer-based competitive immunoassay. The sAA activity was measured using a kinetic assay as described by Schultebraucks, et al. [18].

***Heart rate variability***

Heart rate variability (HRV) was measured using a heart rate monitor and a Polar Electro Oy chest strap (Polar RS800CX; Kempele, Finland). This device is an economic, valid, and reliable alternative to an electrocardiogram [24]. Participants were asked to remain seated in a relaxed position with both feet on the floor and both hands on their lap in a quiet room for 5 min during all measurements. A female researcher was present during the measurements to monitor adherence. HRV was measured at baseline, during the trauma film, and right afterwards. To detect and replace artifacts, the mean values of the previous and following inter-beat intervals were calculated and used as interpolated intervals to replace the abnormal intervals. Default settings were set as “moderate” for the filter power and the minimum protection zone was set at 6 beats per minute (BPM). Filter power indicates the sensitivity cut-off for detecting incorrect deviations from heart rate curve, while the minimum protection zone (with possible settings ranging from 1 to 20 BPM) determined that deviations up to 6 BPM should not be considered measurement errors and should not be corrected. Kubios HRV Version 2.0 (Kubios Oy, Kuopio, Finland) for MATLAB Runtime MCR (R2016b) was used to analyze data. For the statistical analysis the *root mean square of successive differences* (RMSSD) was chosen as the HRV parameter. The RMSSD is a valid indicator of primarily parasympathetic nervous system activity, relatively free of sympathetic inference and respiration rate, when measured at rest and in highly controlled settings. It has therefore been preferred to low-frequency power and low-frequency/high-frequency ratios, which do not explicitly reflect parasympathetic activity but may also reflect sympathetic activity [24].

***Genotyping, quality checks, imputation, and qGWAS***

Genome-wide genotyping of 193 blood-derived DNA samples of self-reported Caucasian ancestry was performed on Infinitum Global Screening array-23 (GSA) MD BeadChip (Illumina, San Diego, CA) at the Human Genotyping Facility (HuGe-F), ERASMUS Medical Center, the Netherlands. Quality checks and genotype imputation were conducted using a previously established GWAS pipeline [25]. Standard quality parameters were applied to exclude individuals with low call rates (< 0.98), variants with high missingness (> 0.02), violations of Hardy–Weinberg equilibrium (*P* < 10^−6^), and deviations of autosomal heterozygosity (| F_het_ | > 0.2). Relatedness testing and principal component analysis (PCA) were done on 91,727 linkage disequilibrium (LD) independent autosomal single nucleotide polymorphisms (SNPs) (minor allele frequency [MAF] > 5%, r^2^ > 0.02) using PLINK [26]. One pair with high relatedness (PI-HAT > 0.2) was identified, of which one randomly selected individual was removed from the data set. Upon visual inspection of PCA plots, we excluded four additional individuals identified as ancestral outliers (see Supplemental Figure 1) resulting in a data set of 188 individuals and 541,619 SNPs. SNP data was then phased and imputed against the public HRC reference panel release 1.1 [27]. After genotype imputation, 8,895,763 SNPs with a MAF > 1% were retained. A quantitative trait GWAS (qGWAS) was conducted on imputed genotype dosages of 147 individuals. The number of intrusions was z-transformed and supplied as quantitative phenotype with the --pheno flag in PLINK. The sample data set was filtered to INFO > 0.6 and MAF > 0.05 to exclude rare or poorly imputed variants. We performed linear regression with five principal components (C1, C2, C3, C4, C11) as covariates to adjust for population stratification. The Manhattan plot of the association results is presented in Supplemental Figure 2, no genomic inflation was observed in the QQ plot (Supplemental Figure 3). As expected, due to the small sample size no genome-wide significant loci were detected. Inspection of the association region plots and INFO scores of the index SNPs suggest that the observed associations are unlikely to be robust. Association results of SNPs in candidate genes (*OXTR, OXT*) were extracted from summary statistics and displayed in Supplemental Table 1.

***Extraction of individual SNPs in the Oxytocin System***

Two SNPs of the oxytocin receptor (rs53576, rs2254298) were identified by literature review of prior studies [28-33]. Genotype probabilities of each SNP were extracted from genotype information of GWAS data to examine their single SNP effects independently of polygenic risks. Hard genotypes were called if the post-imputation genotype probability exceeded 0.8, otherwise they were set to missing. All three variants had a minor allele frequency (MAF) > 0.05 and good imputation quality scores (INFO > 0.8). We calculated the allele dosage for each *OXTR* SNP as the sum of the probability of the AG allele and the twofold probability of the AA allele.

***Polygenic scoring***

Publicly available summary statistics from the psychiatric genomics consortium (PGC) were used as training data sets to construct polygenic risc scores (PRS) for major depressive disorder (MDD) (N_cases_= 135,458; N_controls_= 344,901; [34]), cross-disorder (CRD) (N_cases_= 33,331; N_controls_= 27,888; [35]), schizophrenia (SCZ) (N_cases_= 67,097; N_controls_= 93,459; [36]), and PTSD (females only, N_cases_= 12,954; N_controls_= 73,545; [37]). Obtained summary statistics were LD-pruned and clumped by discarding variants in r^2^ ≥ 0.1 with another more significant variant within a 500 kb window. Indels, strand-ambiguous, and low-frequency (MAF < 5%) variants were removed and only SNPs with high imputation quality (INFO > 0.9) were retained. PRS were computed with PLINK by multiplying the log OR of LD-independent SNPs in the training data sets with the imputation probability for the effect allele in each individual in the present target sample. These weighted values were summed across the genome to obtain individual-level PRS that reflect the genetic propensity towards each trait of interest. To reduce the possibility of inflated type I error rates, no p-value thresholding was conducted. In total, 147 individuals with full phenotypic data had genome-wide PRS for MDD (N_SNPs_= 107,115), PTSD (N_SNPs_= 161,904), CRD (N_SNPs_= 111,412), and SCZ (N_SNPs_= 102,674) for subsequent analyses. The first four principal components were included in each model to control for fine-grained population stratification in the sample.

**Statistical analysis**

*Data preprocessing*

To estimate the *heterogeneous treatment effects* (HTE), the data were preprocessed using dummy coding of categorical variables and predictor variables were checked for zero variance [38]. We also centered and scaled numerical variables by subtracting the mean and dividing by the SD. The highest percentage of missing values per variable was 13% (mean=3%, SD=5%). Missing values were imputed using bagged decision trees [39].

*Hyperparameter tuning*

To estimate the HTE, we trained a GRF model using the “causal_forest” function in the R package GRF [40] and using the internal validation procedure to obtain the optimal combination of the model parameters (i.e., "sample.fraction", "mtry", "min.node.size", "alpha", and "imbalance.penalty") for estimating the CATE (i.e., the ATE conditional on the covariates). We used 555 trees to optimize parameters and set the maximum number of explored hyperparameter combinations to 555. The GRF was trained with 2,051 decision trees as more trees likely increases the accuracy of the results [41]. Trees are split to maximize heterogeneity in the estimated CATE and the parameters are chosen based on estimates in the “out-of-bag” training samples [42-44].

*Overlap assumption*

When examining HTE, the overlap assumption states that there is appropriate overlap in the covariate distributions of the intervention and control groups [45]. Given the randomization procedure, we should not be able to determine the treatment status of an individual based on their covariates [46]. The assumption is checked using a histogram [45].

**Extended results**

**Variable importance**

We found that the oxytocin receptor SNP rs53576 was an important variable in the random forest model to predict heterogeneity in the treatment effect (i.e., the association between the treatment allocation [oxytocin vs. placebo] and the number of intrusive memories [outcome measure]). In the oxytocin group but not the placebo group, the number of intrusions was higher when the allele dosage was lower (Supplemental Figure 5). As shown in Supplemental Figure 6, a post-hoc analysis revealed an effect of the rs53576 GG (p=0.016) and AG polymorphism (p=0.032) on the effect of oxytocin on intrusive memories, but not of the rs53576 AA polymorphism (Supplemental Figure 5). Those participants with the rs53576 GG polymorphism showed significantly more intrusive memories after oxytocin administration than those without the rs53576 GG polymorphism did. In contrast, those with the rs53476 AG polymorphism showed fewer intrusive memories after oxytocin administration than those without this polymorphism did (Supplemental Figure 5). These exploratory effects shown in Supplemental Figure 5 were not significant after correcting with the Wilcoxon test for multiple testing (0.097>p>0.05) [47].

**
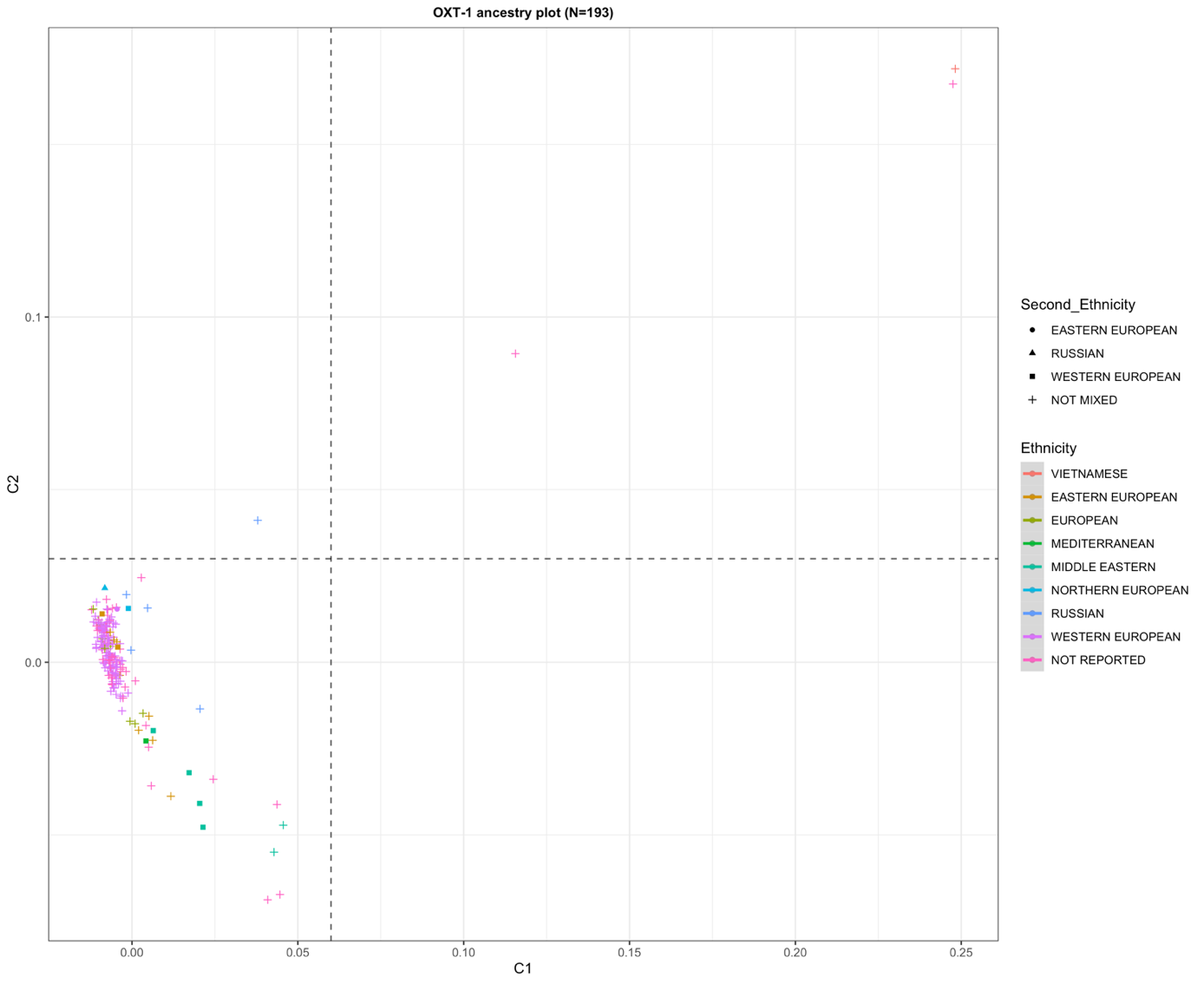
**

Supplemental Figure 1. Principal Components Analysis. Genotype-based population clustering of 193 individuals. Self-reported ancestry is indicated by color and shape (indicating a second ancestry in admixed samples). Dashed lines represent cut-off values for population outlier with C1 > 0.06 (vertical line) and C2 > 0.03 (horizontal line).

**
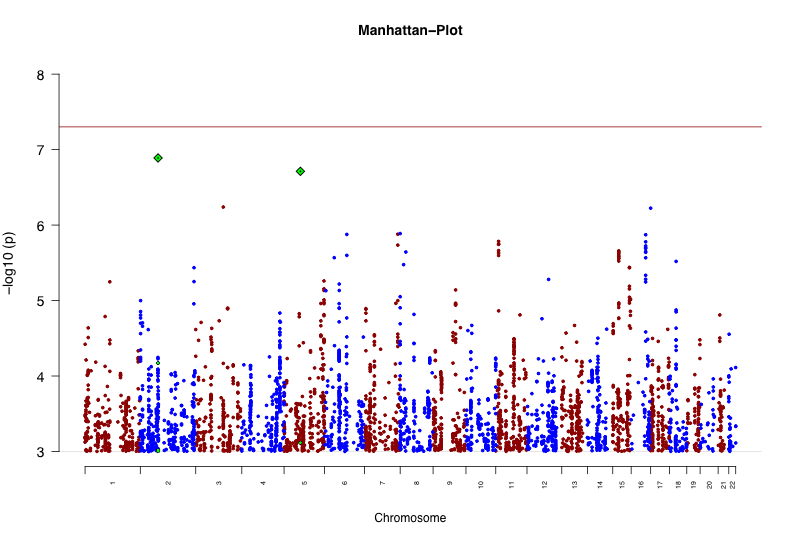
**

Supplemental Figure 2. Manhattan plot for genetic associations with number of intrusions (N=147). The y-axis shows the significance of association (-log_10_(P)) with the intrusion phenotype sorted by chromosome (x-axis). Green diamonds represent index single nucleotide polymorphisms (SNPs) in LD with other SNPs within the same locus (green dots). The red horizontal line indicates the genome-wide significance level at P < 5 x 10^-8^.

**
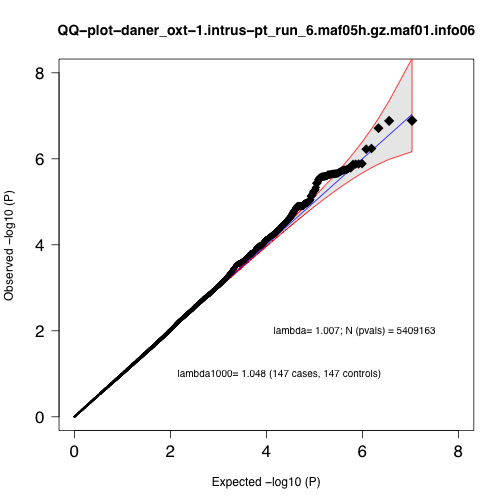
**

Supplemental Figure 3. QQ plot of the qGWAS. Observed –log_10_(P) plotted against expected –log_10_(P) values sorted from smallest to largest –log10P. The blue line indicates a distribution under the null hypothesis with 95% confidence intervals (area shaded in grey).

Supplemental Table 1. Association of SNPs in candidate genes (*OXT*, *OXTR*) with number of intrusions (N=147)

| **CHR** | **SNP** | **BP** | **A1** | **A2** | **FRQ_A1** | **INFO** | **BETA** | **SE** | **P** |
| --- | --- | --- | --- | --- | --- | --- | --- | --- | --- |
| 3 | rs53576 | 8804371 | A | G | 0.3282 | 0.8564 | 0.0072 | 0.1379 | 0.9585 |
| 3 | rs2254298 | 8802228 | G | A | 0.9172 | 0.8637 | -0.2196 | 0.2301 | 0.3416 |

CHR = chromosome; BP = base pair (genomic position in hg19); A1 = effect allele; A2 = non-effect allele; FRQ_A1 = frequency of the effect allele in the sample; INFO = imputation quality score; BETA = regression coefficient; SE = standard error of the regression coefficient; P = p-value; OXT = oxytocin; OXTR = oxytocin receptor.

| **Characteristics** | **Included (n = 201)**  M (SD) or n | **Excluded (n = 19)**  M (SD) or n | **Statistics** |
| --- | --- | --- | --- |
| Age | 23.08 (3.32) | 22.63 (3.30) | U=1741.50, Z = -64, p=0.52 |
| Intake of oral contraceptives | 78 (38.81 %) | 6 (31.58 %) | $\chi$^2^(1)=.44, p=0.51 |
| Current smoker | 61 (30.35 %) | 7 (36.84 %) | $\chi$^2^(1)=.41, p=0.52 |
| BMI | 21.75 (2.49) | 21.54 (2.82) | U = 1859.00, Z = -.19, p=0.85 |
| CTQ | 30.95(6.89) | 33.67(7.32) | U = 1479.50, Z = -1.63, p=0.10 |
| STAI-T | 32.74(6.20) | 35.05(7.02) | U = 1473.50, Z = -1.62, p=0.11 |
| BDI-II | 3.49 (3.32) | 10.05 (7.04) | U = 795.50, Z =-4.23, p<0.001 |
| ERQ reappraisal | 29.29(5.12) | 29.11(3.43) | U = 1832.00, Z = -.29, p=0.77 |
| ERQ suppression | 11.06(3.98) | 12.11(4.96) | U = 1826.00, Z = -.32, p=0.75 |
| Participants who had seen the film before | 16 (7.96 %) | 0 (0.00 %) | $\chi$^2^(1)=1.38, p=0.24 |

Supplemental Table 2: Excluded vs. included

**Note:** M = mean; SD = standard deviation; BMI = body mass index; CTQ **=** Childhood Trauma Questionnaire; STAI-T = State-Trait Anxiety Inventory–Trait subscale; BDI-II = Beck Depression Inventory–Revised; ERQ = Emotion Regulation Questionnaire (subscales reappraisal and suppression).

Supplemental Figure 4. Overlap assumption.


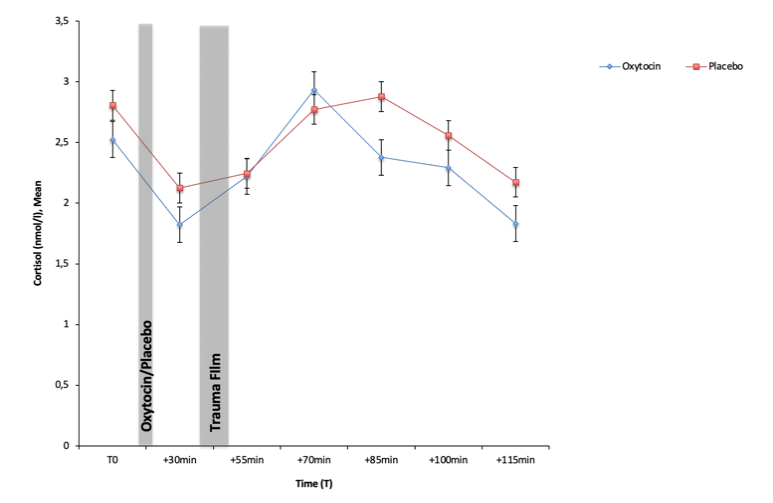

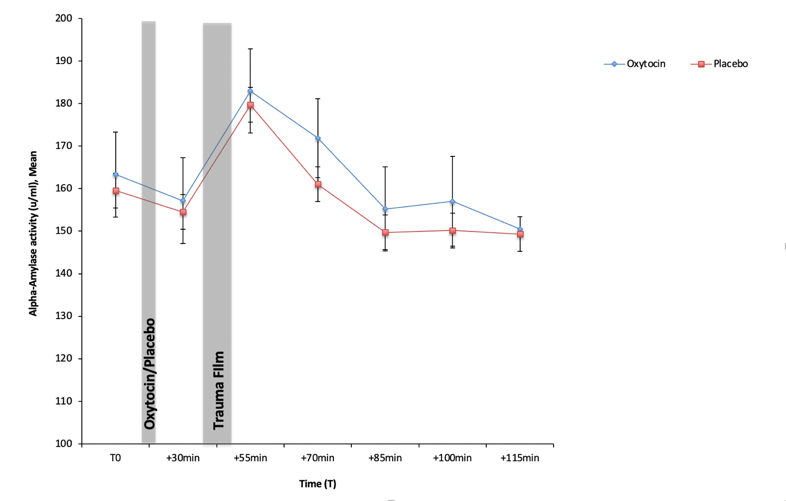


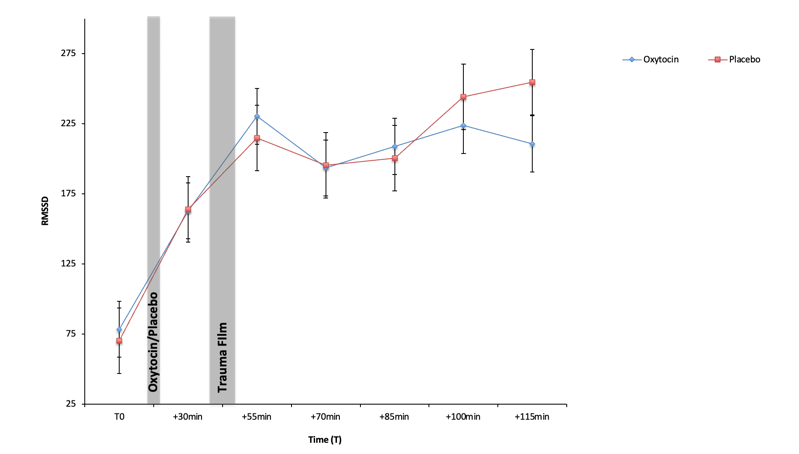


Supplemental Figure 5. Physiological stress response – salivary cortisol (top left), salivary α-amylase activity (top right) and heart rate variability measured as root mean square of successive differences (RMSSD).

Note. Points are means, with standard errors represented by vertical bars.

Supplemental Figure 6. Poisson regression – regression lines (95%CI bands) for the effect of the oxytocin receptor single nucleotide polymorphism (SNP) rs53576 on the number of intrusive memories separately for the treatment group receiving oxytocin (treatment: 1) vs placebo (treatment: 0).

Supplemental Figure 7. Box plots plus significance tests. Oxytocin receptor single nucleotide polymorphism (OXTR SNP) rs53576 below 0.7 were defined as “not present” and above as “present”. OXTR SNP rs53576 GG polymorphism was present in N=71 participants (not present in N=108 and missing in N=22). OXTR SNP rs53576 AA polymorphism was present in N=15 participants (not present in N=164 and missing in N=22). OXTR SNP rs53576 AG polymorphism was present in N=76 participants (not present in N=103 and missing in N=22). We adjusted the p-values for controlling the false discovery rate (fdr) using the R function p.adjust and the Benjamini & Hochberg (1995) correction [47].

Supplemental Figure 8. Coefficient estimates of the step-wise Poisson regression (see Table 2). Significant effects are indicated with asterisks (*<0.05, **< 0.01, ***<0.001). The regression coefficients are plotted with the highest coefficient on top and the vertical red line indicates the intercept of “no effect”.

(a)

(b)

Supplemental Figure 9. Step-wise Poisson regression – regression lines (95%CI bands) for the effect of polygenic risc score (PRS) for post-traumatic stress disorder PTSD (a) and PRS for major depressive disorder (MDD) (b) on the number of intrusive memories separately for the treatment group receiving oxytocin (treatment: 1) vs placebo (treatment: 0).

**Supplemental References**

1 SR Smith. The endocrinology of obesity. Endocrinology and metabolism clinics of north america. 1996;25(4):921-42.

2 MFJ Weingarten, M Scholz, T Wohland, K Horn, M Stumvoll, P Kovacs, et al. Circulating oxytocin is genetically determined and associated with obesity and impaired glucose tolerance. The Journal of Clinical Endocrinology & Metabolism. 2019;104(11):5621-32.

3 N Constantini,AC Hackney. Endocrinology of physical activity and sport. Springer; 2013.

4 JO Tweed, SH Hsia, K Lutfy,TC Friedman. The endocrine effects of nicotine and cigarette smoke. Trends in Endocrinology & Metabolism. 2012;23(7):334-42.

5 H Wittchen, M Zaudig,T Fydrich. Achse I: Psychische Störungen. SKID Strukturiertes Klinisches Interview für DSM-IV. 1997;1.

6 AT Beck, RA Steer,GK Brown. Manual for the beck depression inventory-II. San Antonio, TX: Psychological Corporation. 1996;1:82.

7 K Wingenfeld, C Spitzer, C Mensebach, HJ Grabe, A Hill, U Gast, et al. [The German Version of the Childhood Trauma Questionnaire (CTQ):Preliminary Psychometric Properties.]. Psychother Psychosom Med Psychol. 2010;60(8):e13.

8 A Hofmann, G Fischer,F Koehn. Traumatic antecedents questionnaire (TAQ). Deutsches Institut für Psychotraumatologie, Köln. 1999.

9 C Otte, TC Neylan, N Pole, T Metzler, S Best, C Henn-Haase, et al. Association between childhood trauma and catecholamine response to psychological stress in police academy recruits. Biol Psychiatry. 2005;57(1):27-32.

10 N Breslau, HD Chilcoat, RC Kessler,GC Davis. Previous exposure to trauma and PTSD effects of subsequent trauma: results from the Detroit Area Survey of Trauma. Am J Psychiatry. 1999;156(6):902-7.

11 B Abler,H Kessler. Emotion regulation questionnaire–Eine deutschsprachige Fassung des ERQ von Gross und John. Diagnostica. 2009;55(3):144-52.

12 AN Kaczkurkin, Y Zang, NG Gay, AL Peterson, JS Yarvis, EV Borah, et al. Cognitive emotion regulation strategies associated with the DSM‐5 posttraumatic stress disorder criteria. Journal of Traumatic Stress. 2017;30(4):343-50.

13 L Laux, P Glanzmann, P Schaffner,CD Spielberger. Das state-trait-angstinventar [The state-trait anxiety inventory]. Hogrefe, Göttingen (in German). 1981.

14 RJ McNally, JP Hatch, EM Cedillos, CA Luethcke, MT Baker, AL Peterson, et al. Does the repressor coping style predict lower posttraumatic stress symptoms? Military medicine. 2011;176(7):752-56.

15 EA Holmes,C Bourne. Inducing and modulating intrusive emotional memories: A review of the trauma film paradigm. Acta psychologica. 2008;127(3):553-66.

16 EA Holmes, CR Brewin,RG Hennessy. Trauma films, information processing, and intrusive memory development. J Exp Psychol Gen. 2004;133(1):3-22.

17 A Weidmann, A Conradi, K Groger, L Fehm,T Fydrich. Using stressful films to analyze risk factors for PTSD in analogue experimental studies--which film works best? Anxiety Stress Coping. 2009;22(5):549-69.

18 K Schultebraucks, F Rombold-Bruehl, K Wingenfeld, J Hellmann-Regen, C Otte,S Roepke. Heightened biological stress response during exposure to a trauma film predicts an increase in intrusive memories. Journal of abnormal psychology. 2019;128(7):645.

19 F Rombold, K Wingenfeld, B Renneberg, J Hellmann-Regen, C Otte,S Roepke. Influence of the noradrenergic system on the formation of intrusive memories in women: An experimental approach with a trauma film paradigm. Psychological Medicine. 2016;46(12):2523-34.

20 G Noé, T Bangalter, M Bellucci, V Cassel,A Dupontel. Irreversible. StudioCanal; 2002.

21 EA Holmes, CR Brewin,RG Hennessy. Trauma films, information processing, and intrusive memory development. Journal of Experimental Psychology: General. 2004;133(1):3.

22 A Ehlers, A Hackmann,T Michael. Intrusive re‐experiencing in post‐traumatic stress disorder: Phenomenology, theory, and therapy. Memory. 2004;12(4):403-15.

23 A Arntz, C de Groot,M Kindt. Emotional memory is perceptual. Journal of Behavior Therapy and Experimental Psychiatry. 2005;36(1):19-34.

24 S Laborde, E Mosley,JF Thayer. Heart Rate Variability and Cardiac Vagal Tone in Psychophysiological Research - Recommendations for Experiment Planning, Data Analysis, and Data Reporting. Front Psychol. 2017;8:213.

25 M Lam, S Awasthi, HJ Watson, J Goldstein, G Panagiotaropoulou, V Trubetskoy, et al. RICOPILI: rapid imputation for COnsortias PIpeLIne. Bioinformatics. 2020;36(3):930-33.

26 S Purcell, B Neale, K Todd-Brown, L Thomas, MA Ferreira, D Bender, et al. PLINK: a tool set for whole-genome association and population-based linkage analyses. The American journal of human genetics. 2007;81(3):559-75.

27 S McCarthy, S Das, W Kretzschmar, O Delaneau, AR Wood, A Teumer, et al. A reference panel of 64,976 haplotypes for genotype imputation. Nature genetics. 2016;48(10):1279.

28 LM Sippel, S Han, LE Watkins, I Harpaz-Rotem, SM Southwick, JH Krystal, et al. Oxytocin receptor gene polymorphisms, attachment, and PTSD: Results from the National Health and Resilience in Veterans Study. Journal of psychiatric research. 2017;94:139-47.

29 RG Lucas-Thompson,EA Holman. Environmental stress, oxytocin receptor gene (OXTR) polymorphism, and mental health following collective stress. Hormones and behavior. 2013;63(4):615-24.

30 C Feng, A Lori, ID Waldman, EB Binder, E Haroon,JK Rilling. A common oxytocin receptor gene (OXTR) polymorphism modulates intranasal oxytocin effects on the neural response to social cooperation in humans. Genes, Brain and Behavior. 2015;14(7):516-25.

31 EL Smearman, LM Almli, KN Conneely, GH Brody, JM Sales, B Bradley, et al. Oxytocin receptor genetic and epigenetic variations: association with child abuse and adult psychiatric symptoms. Child development. 2016;87(1):122-34.

32 RP Ebstein, A Knafo, D Mankuta, SH Chew,P San Lai. The contributions of oxytocin and vasopressin pathway genes to human behavior. Hormones and behavior. 2012;61(3):359-79.

33 R Feldman, M Monakhov, M Pratt,RP Ebstein. Oxytocin pathway genes: evolutionary ancient system impacting on human affiliation, sociality, and psychopathology. Biological psychiatry. 2016;79(3):174-84.

34 NR Wray, S Ripke, M Mattheisen, M Trzaskowski, EM Byrne, A Abdellaoui, et al. Genome-wide association analyses identify 44 risk variants and refine the genetic architecture of major depression. Nature genetics. 2018;50(5):668-81.

35 SH Lee, S Ripke, BM Neale, SV Faraone, SM Purcell, RH Perlis, et al. Genetic relationship between five psychiatric disorders estimated from genome-wide SNPs. Nature genetics. 2013;45(9):984.

36 S Ripke, JT Walters, MC O'Donovan,SWGotPG Consortium. Mapping genomic loci prioritises genes and implicates synaptic biology in schizophrenia. MedRxiv. 2020.

37 CM Nievergelt, AX Maihofer, T Klengel, EG Atkinson, C-Y Chen, KW Choi, et al. International meta-analysis of PTSD genome-wide association studies identifies sex-and ancestry-specific genetic risk loci. Nature communications. 2019;10(1):1-16.

38 M Kuhn. The caret R package version 6.0-86. R Foundation for Statistical Computing, Vienna, Austria <https://CRANR-projectorg/package=caret>. 2012.

39 M Kuhn,K Johnson. Applied predictive modeling. Springer; 2013.

40 J Tibshirani, S Athey,S Wager. grf: Generalized Random Forests. R package version 120 <https://CRANR-projectorg/package=grf>. 2020.

41 G Biau,E Scornet. A random forest guided tour. TEST. 2016;25(2):197-227.

42 S Wager,S Athey. Estimation and inference of heterogeneous treatment effects using random forests. Journal of the American Statistical Association. 2018;113(523):1228-42.

43 S Athey,G Imbens. Recursive partitioning for heterogeneous causal effects. Proceedings of the National Academy of Sciences. 2016;113(27):7353-60.

44 S Athey, J Tibshirani,S Wager. Generalized random forests. The Annals of Statistics. 2019;47(2):1148-78.

45 GW Imbens. Nonparametric estimation of average treatment effects under exogeneity: A review. Review of Economics and statistics. 2004;86(1):4-29.

46 DB Rubin. Estimating causal effects of treatments in randomized and nonrandomized studies. Journal of educational Psychology. 1974;66(5):688.

47 Y Benjamini,Y Hochberg. Controlling the false discovery rate: a practical and powerful approach to multiple testing. Journal of the Royal statistical society: series B (Methodological). 1995;57(1):289-300.
